#  Implementing an arts-based intervention for patients with end-stage kidney disease whilst receiving haemodialysis: a feasibility study protocol

**DOI:** 10.1186/s40814-018-0389-y

**Published:** 2019-01-05

**Authors:** Claire Carswell, Joanne Reid, Ian Walsh, Helen McAneney, Helen Noble

**Affiliations:** 1 School of Nursing and Midwifery, Queen’s University Belfast, 97 Lisburn Road, Belfast, Northern Ireland; 2 School of Medicine, Dentistry and Biomedical Sciences, Queen’s University Belfast, Belfast, Northern Ireland

**Keywords:** Art, Feasibility studies, Kidney failure, chronic, Randomised controlled trials as topic, Renal dialysis

## Abstract

**Background:**

End-stage kidney disease is a life-changing illness. Many patients require haemodialysis, a treatment that impacts profoundly on quality of life and mental health. Arts-based interventions have been used in other healthcare settings to improve mental health and quality of life; therefore, they may help address the impact of haemodialysis by improving these outcomes. However, there is a lack of evidence assessing their effectiveness in this population and few randomised controlled trials (RCTs) evaluating the effectiveness of complex arts-based interventions.

**Methods:**

The aims of this study are to establish the feasibility of a cluster RCT of an arts-based intervention for patients with end-stage kidney disease whilst receiving haemodialysis through a cluster randomised pilot study, explore the acceptability of the intervention with a process evaluation and explore the feasibility of an economic evaluation. The study will have three phases. The first phase consists of a cluster randomised pilot study to establish recruitment, participation and retention rates. This will involve the recruitment of 30 participants who will be randomly allocated through cluster randomisation according to shift pattern to experimental and control group. The second phase will be a qualitative process evaluation to establish the acceptability of the intervention within a clinical setting. This will involve semi-structured interviews with 13 patients and three focus groups with healthcare professionals. The third phase will be a feasibility economic evaluation to establish the best methods for data collection within a future cluster RCT.

**Discussion:**

Arts-based interventions have been shown to improve quality of life in healthcare settings, but there is a lack of evidence evaluating arts-based interventions for patients receiving haemodialysis. This study aims to assess the feasibility of a future cluster RCT assessing the impact of an arts-based intervention on the wellbeing and mental health of patients receiving haemodialysis and identify the key factors leading to successful implementation. The hope is this study will inform a trial that can influence future healthcare policy by providing robust evidence for arts-based interventions within the haemodialysis setting.

**Trial registration:**

The trial was prospectively registered on clinicaltrials.gov on 14/8/2018, registration number NCT03629496.

**Electronic supplementary material:**

The online version of this article (10.1186/s40814-018-0389-y) contains supplementary material, which is available to authorized users.

## Background

End-stage kidney disease is the final stage of chronic kidney disease. It is defined by an estimated glomerular filtration rate of < 15 ml/min/1.73 m^2^ [1]. The estimated glomerular filtration rate is the estimated rate of fluid filtration within the glomerulus of the kidney and is the main physiological indicator of renal function. Patients with end-stage kidney disease are poly-symptomatic and experience difficult symptoms including fatigue, pruritus, pain, nausea, sexual dysfunction and muscle weakness, which can profoundly impact quality of life (QoL) [2]. The main treatment modalities for end-stage kidney disease are renal replacement therapies, such as haemodialysis. Haemodialysis is a difficult and time-consuming treatment that requires patients to attend hospital up to three times a week for approximately 4 h each time. During treatment, the patient is connected to a dialysing unit to filter their blood and remove waste products and excess fluid, replacing the role of the kidneys. Patients receiving haemodialysis have lower health-related quality of life (HRQoL) than the general population [3]. Lower HRQoL is associated with increased morbidity and mortality in patients on haemodialysis [4]. Patients receiving haemodialysis also have higher rates of anxiety and depression, with approximately 20–50% of the patient population experiencing depression and/or anxiety [5–9].

Depression is an independent predictor of mortality in haemodialysis populations [10, 11]; depression may result in poor treatment adherence [6, 12], maladaptive health behaviours [13], self-harm and increased suicide risk [14]. Management of end-stage kidney disease involves an extensive and challenging treatment regimen including compliance with dialysis sessions, medications and fluid and diet restrictions [6]. Non-adherence to such regimens can increase mortality risk—missing a single dialysis session each month increases risk of death by 30% [15]. Depression is a contributing factor to non-adherence for patients receiving haemodialysis [12]. Anxiety can also have an impact on the physical health of patients receiving haemodialysis, as anxiety symptoms have a significant relationship with performance status [16, 17]. Therefore, symptoms of both anxiety and depression have a potential impact on the physical wellbeing of patients receiving haemodialysis.

Another collective relationship exists between HRQoL, anxiety and depression in patients receiving haemodialysis. A cross-sectional multi-centre study conducted in Malaysia found that depression, anxiety and stress correlated significantly with HRQoL [8]. Cohort studies within the UK and Ireland also report this association [3]. A systematic literature review found 100% of identified articles showed a statistically significant relationship between anxiety, depression and HRQoL [18]. Clinical depression influences QoL in varying ways, including impacting mood and motivation [19, 20]. As previously described, depression can lead to poor treatment adherence [6], which increases symptom burden. Symptoms experienced by patients with end-stage kidney disease impact mood and increase depressive symptoms [21–23]. The relationship between HRQoL, anxiety and depression is enmeshed, yet anxiety and depression remains underdiagnosed and under-treated in haemodialysis patients [7, 24–26]. One reason for this complex dynamic is the overlap between anxiety, depression and the uremic state; many symptoms of depression and anxiety, such as anorexia, sleep disturbance and sexual dysfunction, are identical to symptoms of uraemia, making it difficult to differentiate anxiety or depression from the clinical picture of end-stage kidney disease [5, 26]. Stigma surrounding depression also contributes to diagnosis and treatment rates, as patients are reluctant to agree to psychiatric assessments [27]. HRQoL, anxiety and depression cannot, therefore, be considered in isolation from each other when caring for patients with end-stage kidney disease.

### Arts-based interventions

The application of arts in health has received recent interest because of its’ potential to improve patient outcomes and reduce costs for the National Health Service (NHS) [28]. Arts-based interventions involve the implementation of arts activities in a healthcare context to deliver a creative experience [29]. They have been shown to improve QoL, symptom burden and mental health [30–32] in a variety of settings, including the medical-surgical setting [32], primary care [33] and cancer care [34], although there is a dearth of evidence exploring their effect in renal populations. Research examining arts-based interventions is small in scale [35], lacks longitudinal follow-up [36] and focuses on receptive interventions such as music listening [30, 31]. There are few studies examining the effect of arts-based interventions in patients with end-stage kidney disease; some studies have examined music listening in patients receiving haemodialysis [37], whilst others have explored the use of arts-in-medicine programs. There is a lack of consistent outcome measures in these studies, but there is some evidence that arts-based interventions could have a wide variety of beneficial effects, such as reducing pain and other unpleasant symptoms [38, 39], improving physiological parameters such as oxygen saturation and respiratory rate [39–41], reducing anxiety [42], improving HRQoL [43] and potentially improving biochemical values such as albumin [43]. However, the research exploring arts-in-medicine programs tends to be observational [43] or exploratory [44, 45], and no randomised controlled trials have been conducted on non-music listening interventions for patients with end-stage kidney disease receiving haemodialysis.

To evaluate the effectiveness of arts-based interventions, rigorous RCTs are necessary. However, there are difficulties associated with RCTs in this arena, particularly around participant recruitment and retention [46]. This is exacerbated in palliative care trials, where participation rates under 50% are common [47] and trials in nephrology also experience problems retaining participants [48]. Methodology in arts-based intervention research needs to be considered due to the lack of RCTs conducted using non-music interventions [31, 36] and the tendency of complex interventions to fail to demonstrate a statistically significant effect [49]. When evaluating complex interventions, an assessment of the intervention’s acceptability for patients and healthcare professionals (HCPs) should be conducted [50]. To explore the acceptability of an intervention, a process evaluation must be conducted. Process evaluations provide an understanding of complex interventions by examining their implementation, mechanism of impact and context [51]. When exploring HCPs’ experiences of arts-based interventions, it is also important to examine the impact on HCPs themselves [31, 36]. This includes consideration of potential negative effects of arts-based interventions on the clinical working environment. Arts-based interventions have potential to interfere with work flow, increase stress and restrict communication between HCPs [52]. Any negative consequences could impact patient safety; therefore, the acceptability of an intervention for HCPs is important to consider [53]. The acceptability of the intervention for patients must also be considered. If an effective intervention is burdensome, patients may not participate and not experience any potential benefits [53].

There is also a need to consider arts-based interventions from an economic perspective, as current guidance on research into arts-based interventions recommends cost-effectiveness or cost-utility is evaluated [54], as the National Institute for Health and Care Excellence (NICE) use cost-effectiveness to inform recommendations for funding healthcare interventions [55]. Whilst there is evidence that suggests arts-based interventions can save the healthcare system money (arts-on-prescription are thought the save the NHS £216 per person) [35], there is a lack of formal economic evaluations within the evidence base [56]. Consequently, it is important to explore the best methods for collecting the data needed for future analysis, to ensure that a formal economic evaluation is feasible within an RCT.

### Objectives

- To assess the feasibility of conducting a definitive randomised controlled trial to evaluate the impact of an arts-based intervention through a cluster randomised pilot study.
- To explore the acceptability of the implementation of an arts-based intervention and randomised controlled trial in a haemodialysis unit for both patients and HCPs.
- To assess the feasibility of conducting a definitive economic evaluation of an arts-based intervention for patients receiving haemodialysis.

## Methods

### Research design

The study will consist of three phases and will utilise a parallel mixed-method design. Phase 1 will involve a quantitative cluster randomised pilot study, phase 2 is a qualitative process evaluation and phase 3 is a feasibility economic evaluation [57].

### Research setting

The research will be conducted in an outpatient haemodialysis unit of a teaching hospital in the Northern Ireland. The unit provides haemodialysis to approximately 120 patients. Participants will be recruited from the unit using convenience sampling.

### Cluster randomised pilot study

#### Participants

Eligibility criteria for patients:

- Age 18 or over
- Able and willing to provide consent
- Receiving haemodialysis (both incident and prevalent haemodialysis patients will be eligible)

There is little consensus on the appropriate sample size for a feasibility study, with guidance ranging from 12 per arm [58] to 50 per arm [59]. This guidance reflects the inconsistent reporting of feasibility studies. A review of pilot and feasibility studies concluded studies were poorly reported and placed an inappropriate emphasis on hypothesis testing [60]. National Institute of Health Research published guidelines highlighting hypothesis testing is inappropriate in feasibility studies [61]; therefore, a power calculation has not been conducted, as hypothesis testing is not an objective of this trial. A sample size of 30 is recommended by the National Institute for Health Research (NIHR) [62] for parameter estimation within an RCT. A university statistician confirmed that a sample of 30 was appropriate to satisfy the research objectives.

#### Recruitment

Eligible patients will be screened by a gatekeeper working in the haemodialysis unit. Screening logs will be used to measure the proportion of patients who are eligible, who are interested in participating and reasons for non-participation [63]. Patients who are eligible will be asked to consent to a first approach by the researcher, who will describe the study and provide the patient with a participant information sheet. The researcher will allow each patient a period of 48 h to consider participation before providing a standardised consent form.

#### Randomisation

Participants will be randomly allocated to the control group, receiving usual care, or the experimental group, receiving usual care plus the arts-based intervention. The control group will be asked not to participate in visual art or creative writing during haemodialysis sessions until data collection is complete. Once data collection has been completed, the control group will receive the same art materials as the intervention group and a facilitated session of art activities whilst on haemodialysis.

Cluster randomisation according to shift pattern (Monday, Wednesday, Friday or Tuesday, Thursday, Saturday) will be used, as this reduces the risk of contamination of the control group. Any identified instances of contamination or deviations from protocol, such as patients attending different shifts and being exposed to the intervention, will be documented in order to inform the best randomisation strategy within a definitive trial. Researchers will aim to recruit an equal number of participants from each shift to ensure both the control and experimental groups contain an equal number of participants [64]. The randomisation procedure will be carried out by a member of staff within the university who is not affiliated with the study. They will toss a coin which will determine what shift pattern will receive the intervention. The allocation will be placed in a sealed envelope that will be opened by the researcher once baseline data collection has been completed.

#### Intervention

The intervention is based on the psychological theory of flow, which posits the existence of a ‘flow state’, a state of optimal experience that results from complete absorption in a task. In order to induce a flow state, the task must present a challenge to the individual that they can overcome through the development of their skills [65, 66]. Qualitative literature has suggested arts-in-medicine programmes, person-centred arts programmes that are delivered within hospitals, can induce the hallmark experiences of a flow state in patients who participate, such as an altered perception of time and reduction in rumination and anxiety [43, 45, 67]. Therefore, the intervention has been modelled on these programmes, with additional structure put in place to allow for assessment of dose and fidelity.

The intervention consists of six 1-hourly art sessions, implemented at the bedside whilst the participant is receiving haemodialysis and facilitated by the researcher. Each participant will receive the sessions over a course of 3 weeks, receiving two sessions a week. This time frame was chosen in consultation with the study’s interdisciplinary advisory group who recommended each participant receive a day off each week to reduce the potential of fatigue influencing participation, in consultation with experts in the field of arts in health who established six sessions was an adequate dose [68], and reviewing previous literature that identified 1 h is the optimal time frame for implementing an art activity [69]. The activities on offer will consist of a selection of discrete choices, either creative writing and visual art, but will involve a person-centred approach that will allow patients to adapt the activities to their interests and their abilities. This person-centred approach is modelled on the arts-in-medicine programmes that have shown evidence of being sustainable in clinical settings over prolonged periods of time [43–45].

Each participant will receive their own individual arts pack that will contain a standardised set of materials that were selected by the study’s interdisciplinary advisory group. The items were selected according to their ease of use and their ability to be implemented without impacting the clinical setting. Each participant will receive:

- Sketch book
- Graphite pencils
- Graphic pens
- Watercolour paints
- Watercolour brush pen with in-built water container
- Colouring pencils
- Drawing board
- Drawing board clip
- Eraser
- Sharpener
- Pencil grip

Individual packs will be provided to each participant to maintain infection control and reduce any issue of cross-contamination between participants. All participants will keep the packs at the end of the study. Each session will involve one to one facilitation to ensure the activities are accessible for the majority of patients, as arteriovenous fistulas and problems with dexterity can limit a person’s ability to use the materials unassisted. Patient preference of activity, materials and engagement will be captured in activity logs by the researcher implementing the intervention.

#### Data collection and management

##### Feasibility measures

The primary outcome of the trial will be the recruitment rate of participants within a single site. Assessing the ability to recruit and retain participants is a common issue explored in feasibility trials and randomised pilot studies [60, 63, 70, 71]. Reasons for non-participation will be collected in screening logs during the recruitment phase. Previous experience with art will be collected in these screening logs to explore whether experience influences recruitment or retention of participants.

Secondary outcomes include the attrition rate of participants over a period of 3 months to assess the feasibility of longitudinal follow-up within a definitive trial. The acceptability of outcome measures will also be assessed by completion rates of clinical outcome measures, missing data, and will be further informed by the parallel process evaluation.

##### Clinical outcome measures

Baseline demographic and clinical data will include age, gender, ethnicity, education, dialysis vintage, frailty and co-morbidities; this will be collected through self-report to establish any differences between control and experimental groups and to identify any factors that may contribute to participation or attrition within the trial.

Due to the wide variety of primary outcomes evaluated in the existing evidence base, it is not clear what the most appropriate primary clinical outcome measure is for a definitive trial; however, assessment of the appropriateness of clinical outcome measures and exploration of potential mechanisms of impact can be explored during feasibility testing to ensure the most relevant clinical outcomes are included in a definitive trial [72]. The most consistently reported improvement in the existing literature base is anxiety [73]; therefore, the Hospital Anxiety and Depression Scale (HADS) will be used; this scale has been validated in patients with end-stage kidney disease [74] and enables exploration of both depression and anxiety independently through a subscale analysis [75].

The Kidney Disease Quality of Life Short Form 36 (KDQoL-SF36) will be used to measure HRQoL; this is a commonly used tool in renal literature, is both valid and reliable and provides the most comprehensive overview of contributing factors to QoL [76]. This questionnaire was selected as it is the most comprehensive HRQoL scale with the ability to explore different subscales [76], and observational studies have suggested that arts-based interventions may result in improvements in the KDQoL-SF 36, improve physiological parameters and help reduce unpleasant physical symptoms of the disease [38, 39, 43, 77].

Arts-based intervention research faces criticism for lack of longitudinal follow-up, which is necessary to assess sustainability and lasting benefits [36]. Participants who are lost to follow-up in longitudinal RCTs of complex interventions tend to be older, diagnosed with a chronic illness and have higher co-morbidity [78], common demographic factors in patients with end-stage kidney disease [79]. Therefore, to establish feasibility of longitudinal follow-up according to attrition rates and assess the impact of participant burden, clinical outcome measures will be collected at baseline, immediately post-intervention, at 6 weeks and 3 months. The follow-up time points within this feasibility study will allow exploration of reasons for attrition and provide participants with an opportunity to familiarise themselves with the outcome measures and assess participant burden for further exploration within the process evaluation.

**Fig. 1:**
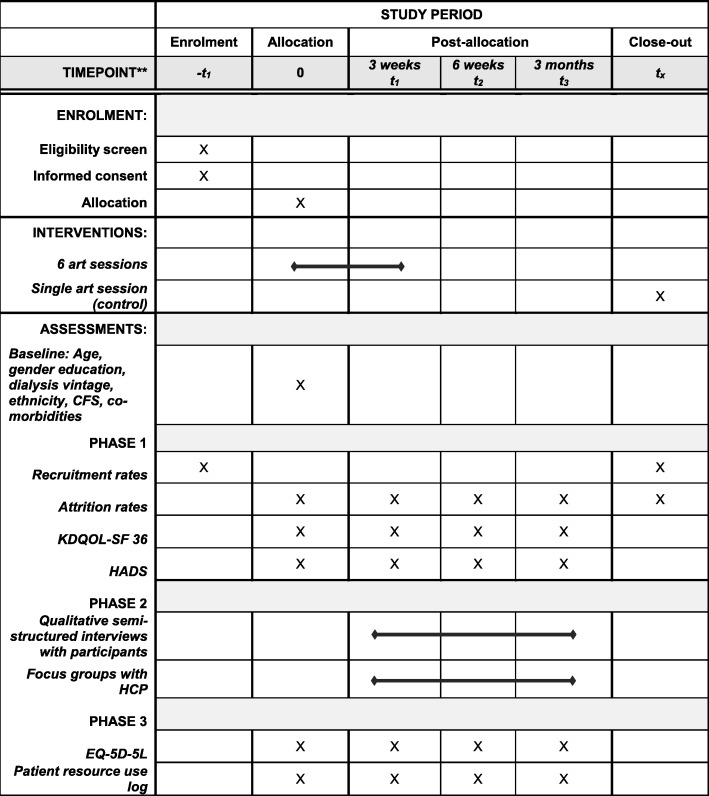
SPIRIT figure illustrating the schedule of enrolment for participants, implementation of interventions and timeline of assessments for phases 1, 2 and 3

The schedule for enrolment, administration of interventions and assessment of outcomes (including those for phase 2 and 3) can be seen in Fig. 1.

#### Data analysis

Data analysis will be conducted using the Statistical Package for the Social Sciences (SPSS v 24). Descriptive statistics will be used to present baseline demographic and clinical data. Categorical data will be presented as frequencies and percentages, whilst continuous data will be presented as means and standard deviations. Recruitment, participation and retention rates will be reported and presented in a CONSORT flow diagram [80]. The proportion of patients who were eligible for recruitment, who consented to participate and who completed the study will be presented with 95% confidence intervals.

Exploratory inferential statistics will be conducted to establish the time needed for data analysis and to explore any potential effects from clustering at the level of shift pattern, but no conclusions of effectiveness of the intervention will be made. Differences in baseline and demographic measures between the clusters will be compared using analysis of variance (ANOVA) for continuous data and *χ*^2^ for categorical data to identify any unanticipated confounding variables introduced by clustering according to shift pattern. As the study is not statistically powered, the results will be interpreted with caution. Independent *t* tests (or Mann-Whitney *U*) will be conducted to compare the average scores of the experimental group and control group. The majority of arts-based intervention research involves pre- and post-test designs; therefore, a repeated measures *t* test (or Wilcoxon matched pairs test) will also be conducted.

### Process evaluation

#### Participants

Participants will include patients and HCPs. The patients recruited will include those who either participated or withdrew from the cluster randomised pilot study. HCPs from the unit will also be recruited into the process evaluation.

Eligibility criteria for HCPs:

- A member of the multidisciplinary healthcare team, including nurses, healthcare support workers, doctors, dietitians, social workers and counsellors who:
  - Has had exposure to the intervention.
  - Has worked in a renal setting for more than 3 months

Familiarity with the context of the clinical environment is needed to inform the acceptability of the intervention [53]. Context includes the social system of the work place, taking into consideration social norms and material resources [53].

#### Recruitment

During the cluster randomised pilot study, patients who have been recruited will be offered the opportunity to participate in the process evaluation. A separate information sheet and consent form will be provided for this phase. Participants from both the intervention and control group will be recruited, and participants will be informed that participation in the process evaluation is not dependent on completion of the cluster randomised pilot study, so participants who decide to withdraw from the feasibility study can be included in the sample.

HCPs will be recruited for the process evaluation by purposive sampling. During the feasibility trial, the researcher implementing the intervention will identify HCPs who are present on the unit during implementation of the intervention. The unit manager, acting as the gatekeeper, will screen the identified HCPs to ensure they meet the inclusion criteria and it is acceptable for the researcher to make the initial approach. Due to managerial and social hierarchies within hospitals, HCPs may feel pressure to participate; therefore, HCPs will be approached by the researcher directly, so the gatekeeper will be unaware of who is participating.

#### Data collection and management

Approximately 13 patients will be recruited into the process evaluation. The principle of 10 + 3 for data saturation outlines a minimum of 10 interviews should be conducted, followed by at least three consecutive interviews that present no new findings [81]. Three focus groups will be conducted with five HCPs per focus group [82, 83]. The dialysis unit in which the study is taking place is small, so the focus group size will be limited by the HCPs available [84, 85]. Data collection will continue until data saturation is reached.

Data collection for the qualitative process evaluation will occur parallel to the cluster randomised pilot study, as this allows adaptation of the intervention to identified concerns during the feasibility stage, as recommended by the Medical Research Council (MRC) guidance [50]. The semi-structured interviews will be conducted with patients in their own home to avoid the power dynamic present in a clinical setting [86]. This approach will be flexible and patients can request a different location. The focus groups with HCPs will take place in a location convenient for all participants. The process evaluation will follow MRC guidance [50] and will focus on the acceptability of the intervention for patients and HCPs, and the interview guides will be informed by the RE-AIM QuEST framework [87]. The interview guide for the semi-structured interviews for patients and focus groups for HCPs can be found in Additional file 1.

#### Data analysis

The semi-structured interviews and focus groups will be conducted by the same researcher and recorded and transcribed verbatim. Inductive thematic analysis will be used to analyse the data collected. Thematic analysis involves identifying and coding central themes within qualitative data through an iterative process [88]. Investigator triangulation will be used to ensure validity of the identified themes [89].

### Economic evaluation

#### Data collection and management

Feasibility studies are not adequately powered to establish effectiveness; therefore, conducting a cost-utility evaluation is not appropriate [90]. However, the feasibility of data collection methods and appropriateness of outcome measures will be evaluated during feasibility testing to inform an economic evaluation within a definitive RCT.

Health and social care costs of participants will be collected using a Patient Service Use Log, a prospective log that captures information on participants healthcare resource use during their time in the study [91]. These will be provided to participants during baseline data collection of the cluster randomised pilot study and collected during follow-up, post-intervention and at 6 weeks and 3 months by the researcher. The outcome of interest is the completion rate for the Patient Service Use Log. The EQ-5D-5 L will be administered to patients in conjunction with clinical outcome measures, pre-/post-intervention and at 6 weeks and 3 months follow-up. The outcome of interest is the acceptability of the EQ-5D-5 L for use within an economic evaluation, as determined by completion rates, missing data and assessment within the process evaluation. The EQ-5D-5 L is recommended by NICE for deriving utility values for the calculation of quality-adjusted life years in economic evaluations [92]. Whilst it is not anticipated that arts-based interventions can improve all items of the EQ-5D-5 L, there is evidence that suggests they may help patients manage pain [38], improve dexterity [93], reduce depression and anxiety [94] and subsequently improves motivation and engagement in daily activities [31, 95, 96].

#### Data analysis

A cost-consequence analysis is recommended by the NIHR for feasibility studies [97]. Costs and outcome measures will be presented using descriptive statistics, including means and 95% confidence intervals to show a general overview of the economic consequences of the intervention. Completion rates and missing data for the Patient Service Use Log and the EQ-5D-5 L will be presented as frequencies to assess the feasibility of data collection.

## Progression criteria

Progression to a definitive RCT will be determined by recruitment rates [98] and the acceptability of the intervention for patients and staff. Acceptability will be evaluated through the qualitative process evaluation, as it is recommended that both quantitative and qualitative data are used when determining the feasibility of a randomised controlled trial [50, 72]:

- 75–100% of the target sample size recruited from a single site will result in progression to a definitive RCT.
- 50–74% of the target sample size recruited from a single site will result in progression to a definitive trial after reviewing the protocol and data from the process evaluation, and making appropriate amendments to address barriers to recruitment.
- 25–49% of the target sample size recruited from a single site will result in progression to a definitive trial after reviewing the protocol with input from potential co-applicants to ensure that the protocol is modified to enhance recruitment rates.
- Less than 25% of the target sample size recruited from a single site will probably result in the trial not progressing, unless a significant modifiable barrier is identified within the process evaluation.
- More than 20% attrition rate [99] from the recruited sample will result in revision of the protocol and data from the process evaluation, and appropriate amendments will need to be made to address barriers to retention of participants, prior to progression to a full trial.
- Progression to a full trial will be contingent on the acceptability of the intervention for both patients and HCPs regardless of recruitment rates. This will be explored within the qualitative process evaluation [87]. Any necessary modifications identified will be made prior to progression to a definitive trial.
- The use of the outcome measures in a definitive trial will be contingent on the acceptability of the questionnaires, which will be informed by completion rates and through the qualitative process evaluation. Any necessary modifications, such as removing questionnaires or using only certain subscales, will be made prior to progression to a definitive trial.

## Discussion

Whilst patients with end-stage kidney disease receiving haemodialysis may stand to benefit from arts-based interventions, the lack of RCTs limits the ability of the evidence base to influence healthcare policy and enable access to these interventions. Arts-based interventions also constitute a complex intervention according to MRC guidance, and therefore, additional factors outside of efficacy, including the context of implementation, acceptability for patients and acceptability for HCPs, also need to be considered to inform optimal strategies for implementation in clinical practice. This article has outlined a protocol to develop an arts-based intervention for patients with end-stage kidney disease whilst receiving haemodialysis and to assess the feasibility of a parallel cluster randomised controlled trial and economic evaluation. This study will provide a foundation from which a definitive RCT protocol can be developed that can assess the effectiveness of highly complex arts-based interventions for patients with end-stage kidney disease receiving haemodialysis, within a framework that can be used to inform healthcare policy.

### Limitations

One limitation of this protocol is the lack of differentiation of roles. As a consequence of limited resources, the development of the intervention, the implementation of the intervention, data collection and data analysis will be conducted by a single research team. Ideally, these roles would be distinct and performed by separate teams, as outlined in the MRC guidelines [50]. Any future definitive trial should have separate teams implementing the intervention, collecting the quantitative data and collecting the qualitative data for a process evaluation to reduce the risk of bias. It is also recommended that in any future process evaluation, multiple team members should transcribe and thematically analyse qualitative data. Another limitation is the lack of attention placebo provided to the control group [100]; whilst no hypothesis testing is being conducted to establish the efficacy of the intervention, attention placebo control may influence retention of participants in the comparator group. Implementation of two different interventions is not feasible within the restricted resources of this study; however, the process evaluation may help inform the elements needed in a future attention placebo control group within a definitive RCT. An additional consideration that will inform a definitive RCT is the need for multiple clusters to control for between-cluster variation. Whilst the size of this study restricted randomisation to only two clusters, a definitive trial will likely require multiple sites to ensure an adequate number of clusters.

## Additional file

Additional file 1: Process evaluation interview guide for experimental group. (DOCX 21 kb)

## Abbreviations

HCP: Healthcare professional
HRQoL: Health-related quality of life
MRC: Medical Research Council
NHS: National Health Service
NICE: National Institute for Health and Care Excellence
NIHR: National Institute for Health Research
QoL: Quality of life
RCT: Randomised controlled trial
