# Supplementary material for: Implementing an arts-based intervention for patients with end-stage kidney disease whilst receiving haemodialysis: a feasibility study protocol
Source: Pilot Feasibility Stud. 2019 Jan 5;5:1. doi: 10.1186/s40814-018-0389-y (PMC6320589; doi:10.1186/s40814-018-0389-y)
Supplement: Supplementary file 1 — Process evaluation interview guide for experimental group. (DOCX 21 kb) [file 40814_2018_389_MOESM1_ESM.docx]

# Interview Guide- Experimental Group

The purpose of this interview is to gain a greater understanding of your experience and opinion of the arts activities that you participated in during the trial. There are a few areas I would like discuss about this experience, both about the activities and different aspects of the study. If you would like to stop the interview at any time just let me know and it will be stopped. We can then start again when you are ready.

How would you normally occupy your time while receiving haemodialysis?

Reach

What were your first thoughts and feelings about participating in art while receiving haemodialysis?

Prompts:

- Did you have previous experiences/interest in art?
- What were your expectations? Did you have any concerns or worries?

Effectiveness

How did participating in art make you feel?

Prompts:

- How do you normally feel during haemodialysis sessions?
- How did you feel during the art-making sessions?
- How did you feel after the art-making sessions?
- Did you interest in/experience of art participation change at all during the trial?
- Do you feel participating in the arts has impacted your life in any other/ unexpected way? Communication/ Relationships/ Health behaviours/ Coping strategies/ Compliance with dialysis or other parts of the treatment
- Would you recommend arts-based activities to others?

Adoption

What made you want to take part and why did you remain in the study?

Prompts:

- Were there any benefits you experienced that made you continue to participate?
- Were there any difficulties that made you feel less motivated to participate?
- Do you feel your treatment/symptoms impacted on your ability to take part? Why/Why not?

Implementation

What did you think about the arts activities?

Prompts:

- What did you think about the selection of art-making materials on offer?
- What did you think about the length of the intervention?
- What did you think about the way the art was facilitated?
- Is there anything you particularly liked about the activities?
- Were there any changes you think should be made to the activities? How can we improve this intervention?
- Was there anything you found particularly helpful to completing the activities? Facilitation/Prompts/ Interest of staff/ Display of completed work

What did you think about the questionnaires?

Prompts:

- Do you feel they covered experiences and symptoms that are important to you?
- How did you feel while completing them? Did you find them difficult/easy to complete?
- Was there anything you found helped you complete the questionnaires? i.e. healthcare staff/researcher facilitation/carer or family input
- Do you feel there is anything else we should be measuring?

Maintenance

Have your thoughts and feelings about art changed since taking part?

Prompts:

- How would you feel about arts being provided as a resource within the haemodialysis?
- Have you participated in art since the intervention? In what way?

Is there anything else you would like to tell me about your experience with the arts-based activities?

# Interview Guide- Control Group

The purpose of this interview is to gain a greater understanding of your experience and opinion of the study you participated in. There are a few areas I would like discuss about this experience. If you would like to stop the interview at any time just let me know and it will be stopped. We can then start again when you are ready.

How would you normally occupy your time while receiving haemodialysis?

Reach

What were your first thoughts and feelings about participating in the research?

Prompts:

- Have you had any previous experiences with research?
- What were your expectations? Did you have any concerns or worries?
- Did an interest in art influence your decision to participate in the study?
- How did you feel about randomisation?

Effectiveness

- Not Applicable

Adoption

Why did you remain in the study?

Prompts:

- Were there any benefits you experienced that made you continue to participate?
- Were there any difficulties that made you feel less motivated to participate?
- Do you feel your treatment/symptoms impacted on your ability to take part? Why/Why not?
- How would you feel about having the opportunity to take part in arts activities whilst receiving haemodialysis?

Implementation

How did you feel about being in the control group?

Prompts:

- Did you find it difficult to participate in research without receiving the intervention?
- How did you feel about knowing that other participants were receiving the intervention while you were not?

What did you think about the questionnaires?

Prompts:

- Do you feel they covered experiences and symptoms that are important to you?
- How did you feel while completing them? Did you find them difficult/easy to complete?
- Was there anything you found helped you complete the questionnaires ? ie. healthcare staff/researcher facilitation/carer or family input
- Do you feel there is anything else we should be measuring?

Maintenance

Have your thoughts and feelings about art changed since taking part?

Prompts:

- How would you feel about arts being provided as a resource within the haemodialysis?
- Have you participated in art since the trial started? In what way?

Is there anything else you would like to tell me about your experience with the research process?

# Focus Group Interview Guide- Healthcare Staff

The purpose of this focus group is to gain a greater understanding of your experience and opinion of the arts activities and research processes that took place on the haemodialysis unit where you work. There are a few areas I would like discuss about this experience, both about the activities and different aspects of the study. If you would like to stop the interview at any time just let me know and it will be stopped. We can then start again when you are ready.

***Reach***

What were your first thoughts and feelings about an arts-based intervention being implemented in the haemodialysis unit where you work?

Prompts:

- Did you think patients would enjoy it?
- Did you think there would be many people willing to participate?
- Were there particular patients you thought would be interested/would benefit? Why?
- Did you have previous experiences/interest in art?
- What were your expectations? Did you have any concerns or worries?

How did you feel about being asked to recruit patients into the study?

Prompts:

- Did you feel comfortable asking patients to participate?
- Did you feel like patients were interested in the study?
- Did you feel that you had enough information to explain the study or would you have preferred more guidance?
- Is there anything you feel should change about how patients are approached for research?

***Effectiveness***

How do you think the arts activities impacted the patients?

Prompts:

- Did you notice a change in the patients participating in the arts activities?
- Do you think the arts activities impacted on their treatment – i.e. compliance? Communication with professionals?
- Do you feel the arts activities were beneficial for patients?

Did the arts activities impact on your relationship with patients?

Prompts:

- Do you feel the activities were a facilitator or barrier to communication with patients?
- Do you feel the arts provided insight into patient experiences?

***Adoption***

How would you describe the level of engagement of patients participating in the study?

Prompts:

- Do you feel patients were motivated to engage in these activities?
- Do you feel clinical factors, such as symptoms, treatment or co-morbidities, impacted on participant’s level of engagement?

***Implementation***

What did you think about the arts activities and how did they impact on the clinical environment?

Prompts:

- What did you think about the selection of art-making materials on offer? Were they appropriate for the clinical environment?
- What did you think about the length of the intervention?
- What did you think about the way the art was facilitated?
- Is there anything you particularly liked about the activities?
- Were there any changes you think should be made to the activities to make them more suitable for the clinical environment?
- Were there any barriers or facilitators to implementation?

***Maintenance***

Have your thoughts and feelings about art changed since the trial took place?

Prompts:

- How would you feel about arts being provided as a resource within the haemodialysis?
- Have you participated in art since the intervention? In what way?

Is there anything else you would like to tell me about your experience with the arts-based activities or the research process?
